# Study on the Effect of Quasi-Radical Lesion Resection on the Quality of Life of Patients With Advanced Hepatic Alveolar Echinococcosis

**DOI:** 10.3389/fsurg.2021.821373

**Published:** 2022-01-21

**Authors:** Jide A, Jinping Chai, Wenlu Guo, Shunyun Zhao, Hao Wang, Xiangren A, Jinyu Yang

**Affiliations:** ^1^Medical College of Soochow University, Suzhou, China; ^2^Department of Hepatic Hydatidosis, Qinghai Provincial People's Hospital, Xining, China; ^3^Department of Internal Medicine-Cardiovascular, Qinghai Provincial People's Hospital, Xining, China; ^4^Qinghai Province Key Laboratory of Laboratory Medicine, Department of Clinical Laboratory, Qinghai Provincial People's Hospital, Qinghai Clinical Medical Research Center, Xining, China; ^5^Department of Hepatic Hydatidosis, Qinghai Provincial People's Hospital, Xining, China; ^6^Intensive Care Unit, Qinghai Provincial People's Hospital, Xining, China

**Keywords:** hepatic alveolar echinococcosis, quasi radical lesion resection, radical lesion resection, quality of life, total survival time

## Abstract

**Objective:**

To retrospectively analyze the effects of radical lesion resection and quasi radical lesion resection on the quality of life of patients with advanced hepatic alveolar echinococcosis.

**Methods:**

Through the existing HIS system of Qinghai Provincial People's Hospital, 104 patients with hepatic alveolar echinococcosis who underwent surgical treatment in our hospital from January 2012 to December 2017 and completed the quality of life questionnaire were selected as the research objects. The above cases were divided into radical group (*n* = 51) and quasi-radical group (*n* = 53) according to different surgical methods (degree of radical cure). The quality of life of patients with hydatidosis was measured by interview or telephone follow-up. The preoperative indexes, intraoperative conditions and postoperative recovery indicators of the two groups were observed, such as Child-Puhg grade, PNM classification, scope of hepatectomy, intraoperative bleeding, Clavien grade, incidence of complications, 5-year recurrence rate and total score of quality of life and so on.

**Results:**

There was no significant difference between the two groups in general data such as age, gender, hydatid size, Child-Puhg grade and preoperative liver function (*P* > 0.05). However, there was a statistically significant difference in PNM classification between the two groups (*P* < 0.05). There were significant differences in intraoperative bleeding, postoperative liver function recovery, Clavien grade of complication severity and 5-year recurrence rate between the two groups (*P* < 0.05). There was no significant difference in postoperative quality of life between the two groups (*P* > 0.05).

**Conclusion:**

For patients with advanced hepatic alveolar echinococcosis whose objective cannot be achieved by conventional hepatectomy, quasi-radical resection of the lesion can not only reduce the risk and difficulty of surgery, but also the quality of life of the patients may be as good as that of radical resection.

## Introduction

Hepatic alveolar echinococcosis (HAE) is a common parasitic disease caused by multilocular Echinococcus infection (1–3), although it is a benign parasitic disease, its biological behavior is malignant, so it is called “Hydatid Cancer.” Hepatic alveolar echinococcosis shows a similar pattern to malignancies in terms of radiologic and clinical features. For this reason, oncological surgical principles should be applied during the resection of hepatic alveolar echinococcosis. The gold standard surgical treatment is resection with negative surgical margin (4). Hence radical lesion resection is the first choice for the treatment of advanced HAE (5, 6). For patients with intermediate and advanced stages, radical resection is still the only effective means of surgical treatment (7). However, radical leision resection with the intent of negative margins (R0) may lead to serious complications such as bleeding and liver failure (8). In addition, many domestic and foreign research results also show that the rate of radical resection of alveolar hydatid disease is still low (9, 10). Quasi-radical lesion resection is an operation in which the lesion resection range reaches more than 95% and the residual hydatid lesion is inactivated after electrocoagulation. This procedure is an alternative operation for patients with end-stage hepatic alveolar echinococcosis who cannot undergo radical lesion resection and whose family members refuse to undergo *in vitro* hepatectomy and autologous liver transplantation. Studies have shown that the survival time and quality of life (QoL) of patients with HAE can be significantly prolonged after radical resection of the lesion (11). However, our follow-up data showed that the QoL of patients after quasi-radical lesion resection were not particularly poor. At present, the long-term efficacy of patients with HAE at home and abroad mainly focuses on the recurrence rate of lesions, but there are few studies on the impact of postoperative QoL. QoL is a concept closely related to the nature of disease, patients' cultural background and psychological factors (12–15). Guo Min (16) from Xinjiang Medical University developed the first evaluation index system of QoL of patients with hydatid surgery in China. This scale was in line with the humanistic beliefs, geographical environment and social factors of the Qinghai-Tibet Plateau, and had high credibility for hydatid patients in Xinning region. Therefore, 104 patients with HAE who underwent surgical treatment and completed follow-up in Qinghai Provincial People's Hospital were selected as the research objects in this paper. The relationship between surgical methods and QoL was discussed as the entry point, and the efficacy of various surgical methods was evaluated.

## Data and Method

### Basic Information of Patients

A total of 385 patients with HAE who received surgical intervention in Qinghai Hydatid Diagnosis and Treatment Center from January 2012 to December 2017, and completed the QoL questionnaire were selected as the study subjects. Inclusion criteria: All patients met the WHO-TWGE PNM (17) diagnostic criteria of intermediate and advanced HAE(P3-4N1M0). All patients underwent surgical treatment of liver lesions in Qinghai Province Hydatid Disease Diagnosis and Treatment Center. All patients underwent liver surgery for the first time. All patients were confirmed as multilocular echinococcosis or alveolar echinococcosis by postoperative pathology. Patients who got in touch and agreed to be investigated, and who signed an informed consent form and completed a QoL measurement scale. Exclusion criteria: Patients who underwent radiofrequency ablation or microwave ablation and died after critical rescue. Patients with extrahepatic echinococcosis. Patients who could not complete follow-up due to refusal or inability to contact were excluded. After screening through the above criteria, 104 patients who met the requirements finally were enrolled in the group and the general information, clinical data and follow-up data of the enrolled patients were collected. The patients were contacted and the contact information of patients was obtained by querying medical records. The QoL of patients with HAE was investigated by interview for those patients who agreed to face to face interview or was investigated by telephone follow-up for other patients who were not convenient for interview.

### Research Object Grouping and Observation Indicators

The above cases were divided into radical lesion resection group (51 cases) and quasi-radical lesion resection group (53 cases), which were referred to as radical group and quasi-radical group. The basic data, intraoperative conditions and postoperative recovery indicators of the two groups of patients were compared. Basic information included age, sex, comorbidities, Child-Pugh grade, PNM classification and preoperative liver function indicators (alanine aminotransferase ALT, aspartate aminotransferase AST, total bilirubin TBIL, direct bilirubin DBIL). Intraoperative information included: Scope of hepatectomy, Intraoperative bleeding and Intraoperative blood transfusion Postoperative recovery measures included postoperative liver function indexes, postoperative complications, 5-year recurrence rate and QoL score.

### Preoperative Preparation

After admission to the hospital, 104 patients underwent contrast-enhanced computed tomography (CT) and CT angiography (CTA) of the head, chest, and abdomen; enzyme-linked immunosorbent assay (Diagnostic Kit for IgG Antibody to Hydatid, ELISA brand is HAI TAI and from Zhuhai special economic zone haitai biopharmaceutical Co. LTD) for the hydatid; and eight tests for infection (Including HBsAg, HBsAb, HBeAg, HBeAb, HBcAb, HCV-Ab, HIV-Ag/Ab, and TPAb). Metastasis of AE to the brain, lung, and other organs was excluded before surgery. The liver reserve function, residual liver volume, and the relationships between the lesion and the blood vessels and bile ducts were evaluated.

There were 6 patients with jaundice in this study, including 6 patients in the quasi-radical lesion resection group with total bilirubin levels ranging from 87.6 to 236.0 umol/L. However, the preoperative Child-Pugh grade of the above cases was grade B, and Among them, 5 patients underwent HAE abscess drainage and hepatocentesis biliary drainage simultaneously. The remaining 1 patients were treated with hydatid necrotic cavity puncture drainage after percutaneous liver puncture biliary drainage failed because intrahepatic bile duct dilation was not obvious. All 6 patients were treated with hepatoprotective drugs, and the liver reserve function was evaluated again after the total bilirubin level returned to normal, and the Child-Pugh grade of all patients was A.

### Surgical Methods and Indications

Radical lesion resection: The range of liver resection should be more than 1.0 cm above the edge of hydatid lesion, and the surgical margin should reach R0 resection. Indications: According to the WHO Guidelines for Diagnosis and Treatment of Echinococcosis (17), patients who are diagnosed with hepatic alveolar echinococcosis and whose lesions are larger than 5.0 cm in diameter and can tolerate surgery should undergo radical resection.

Quasi-radical lesion resection: More than 95% of the hydatid lesions were removed surgically, and only a few lesions were retained on the surface of important hepatic pipes or surrounding organs, and the remaining lesions were electrocoagulation or argon cauterization until the lesions were inactivated. Indications: the scope of lesion invasion is large and the degree of invasion is serious. Conventional hepatectomy can not achieve the purpose of radical cure. It is predicted that the patient can not tolerate the extended operation, and the patient and his family refuse to accept artificial material implantation or autologous liver transplantation.

### Drug Treatment and Follow-Up After Discharge

The patient started to orally take albendazole after discharge with the daily dose of 15 mg/Kg. Medicine was discontinued after 2~3 weeks, and indicators of liver functions were tested. If indicators of liver functions were normal, then medicine was continued after 2~3 days, but if liver damage was severe, it was recommended to have hepatoprotective therapy after discontinuing the medicine. Next cycle of drug-assisted therapy continued after all indicators of liver functions recovered to normal levels. Lifetime use of albendazole was recommended for patients in the quasi-radical resection group and for patients in the radical resection group were recommended for at least 2 years (17), after which the decision to take albendazole was based on review (18). Follow-up visits were made every 6 months within 2 years and every 12 months after 2 years. The review included enzyme-linked immunosorbent assay (ELISA), liver and kidney function tests, abdominal ultrasound or abdominal computer tomography (CT scan of the whole abdomen every 12 months). Diagnosis of hydatid recurrence: imaging examination found hydatid lesions in the liver, and hydatid ELISA was positive. According to WHO Guidelines for Diagnosis and Treatment of Echinococcosis (17), including drugs, reoperation and comprehensive treatment, the treatment plan should be selected according to the specific conditions of the lesions and patients.

### Survey Tool

In this study, the QoL of HAE patients was evaluated from several fields, including physiology domain, psychology domain, independence domain, environment domain and social relationship domain, using the QoL scale developed by Xinjiang Medical University. Each item has 5 options, which are numbered from 0 to 4 points in turn. The reverse item is scored in reverse order, and the total score is 84 points. The higher the score, the better the QoL, and vice versa.

### Statistical Methods

EpiData 3.0 was used to establish a database, and the data of the questionnaire were recorded in parallel. Experimental data were processed by SPSS 22.0 statistical software. The measurement data conforming to normal distribution were expressed as mean±standard deviation, and the independent sample *t*-test was used for comparison between the two groups. P50 (P25, P75) was used for measurement data of non-normal distribution. Wilcoxon rank sum test was used for comparison between two groups. The counting data were analyzed by chi-square test of four-grid table or row × list. Test level *a* = 0.05.

## Results

### The Basic Data of Patients in the Quasi-Radical Group and the Radical Group Were Compared

There was no significant difference in age, sex, hydatid size, complications, Child-Pugh grade and preoperative liver function index between 2 groups (*P* > 0.05). However, there was a statistically significant difference in PNM classification between the two groups (*P* < 0.05). as shown in Table 1.

**Table 1:** Comparison of basic data between the two groups.

**Indices**		**Quasi-radical group(*n* = 53)**	**Radical group (*n* = 51)**	***P***
Age (years)		35 (27, 44)	40 (28, 45)	0.387
Gender	Male	21	22	0.716
	Female	32	29	
Hydatid size (centimeter)		12.08 ± 3.04	11.18 ± 3.43	0.899
Preoperative complication	Yes	25	16	0.099
	No	28	35	
Jaundice		6	0	
HBV		10	9	
HCV		0	1	
Cholecystitis		0	3	
Peritonitis		2	1	
Old pulmonary tuberculosis		1	1	
Renal tuberculosis		2	0	
Anemia		0	1	
Ascites		4	0	
Child–Pugh grade	A	25	30	0.234
	B	28	21	
PNM classification	P3N0M0	10	15	0.001
	P3N1M0	8	21	
	P4N0M0	17	10	
	P4N1M0	18	5	
Preoperative TBIL (umol/L)		13 (10, 19)	12 (10, 15)	0.174
Preoperative DBIL (umol/L)		5 (4, 8)	5 (3, 8)	0.095
Preoperative ALT (U/L)		29 (20, 43)	27 (19, 36)	0.468
Preoperative AST (U/L)		19 (14, 31)	25 (17, 32)	0.123

### Comparison of Intraoperative Situation and Postoperative Recovery Between Quasi-Radical Groups and Radical Groups

There was no significant difference in Scope of hepatectomy and postoperative complication rate between the two groups (*P* > 0.05). Intraoperative bleeding, Intraoperative blood transfusion, postoperative liver function indexes (AST, ALT) and Clavien grade were compared between the two groups, and the differences were statistically significant (*P* < 0.05). In other words, patients in the radical surgery group had advantages of less intraoperative blood loss, faster recovery of postoperative liver function and lower severity of complications, as shown in Table 2.

**Table 2:** Comparison of intraoperative conditions and postoperative recovery between the two groups.

**Indices**		**Quasi-radical group (*n* = 53)**	**Radical group (*n* = 51)**	***P***
Scope of hepatectomy	Lobectomy of liver	33	25	0.194
	Hemihepatectomy	13	21	
	Enlarged hemihepatectomy	7	5	
Intraoperative bleeding(milliliter)		1,000 (500, 1,200)	400 (300, 800)	0.001
Intraoperative blood transfusion(milliliter)		1200 (400, 1,415)	0 (0, 800)	0.001
Postoperative ALT (U/L)	1d	380 (259, 471)	285 (181, 407)	0.031
	3d	183 (116, 301)	153 (98, 246)	0.113
	5d	86 (53, 137)	71 (43, 101)	0.175
Postoperative AST (U/L)	1d	349 (232, 557)	316 (201, 435)	0.120
	3d	98 (52, 188)	66 (44, 127)	0.073
	5d	40 (26, 56)	34 (23, 44)	0.047
Clavien grade	0	12	20	0.002
	1	13	18	
	2	13	11	
	3	15	2	
Postoperative complications	Yes	41	31	0.067
	No	12	20	
Pleural effusion		18	13	
Pleural effusion with ascites		5	8	
Pleural effusion with bile leakage		0	2	
Liver insufficiency		0	8	
Pleural effusion with biliary leakage and abdominal infection		7	0	
Pleural effusion with biliary leakage, jaundice, abdominal infection, abdominal abscess, localized peritonitis		7	0	
Gastrointestinal hemorrhage		4	0	

### Comparison of Total Score of QoL and 5-Year Recurrence Rate Between Quasi-Radical Group and Radical Group

**Figure 1:**
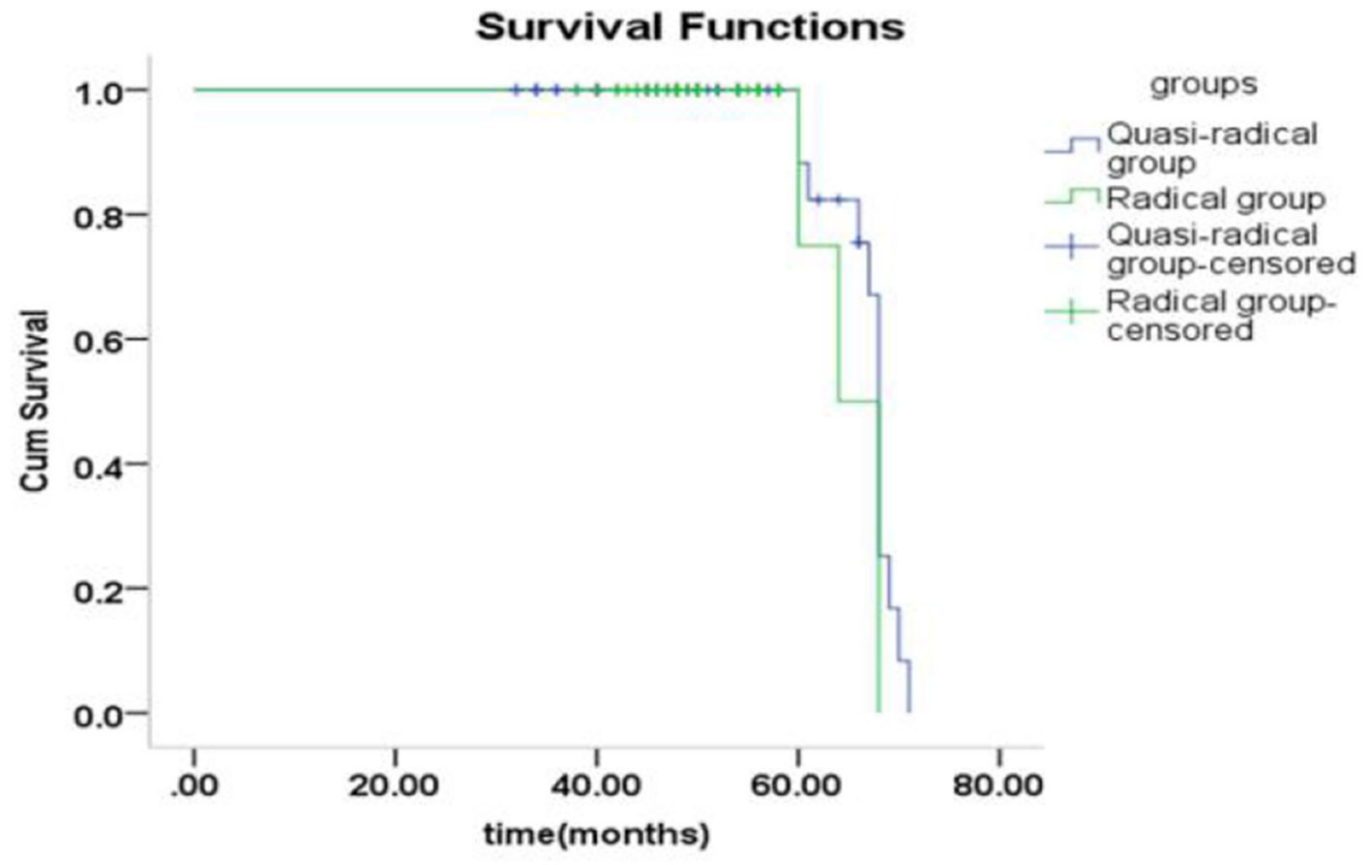
The survival analysis of the two groups of patients with different surgical methods.

**Figure 2:**
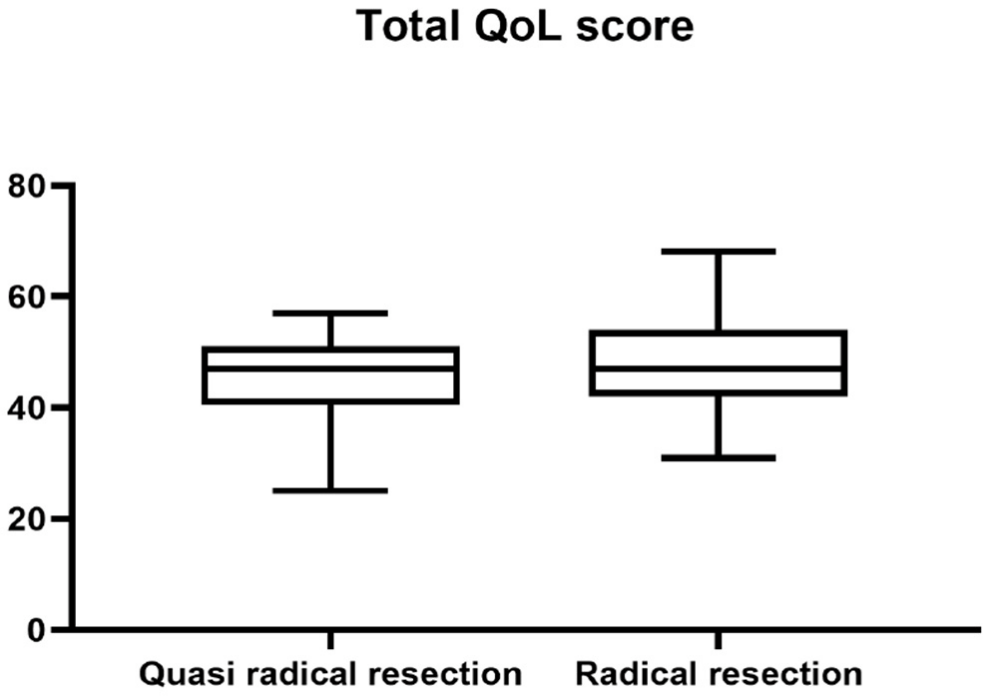
Total QoL score.

**Figure 3:**
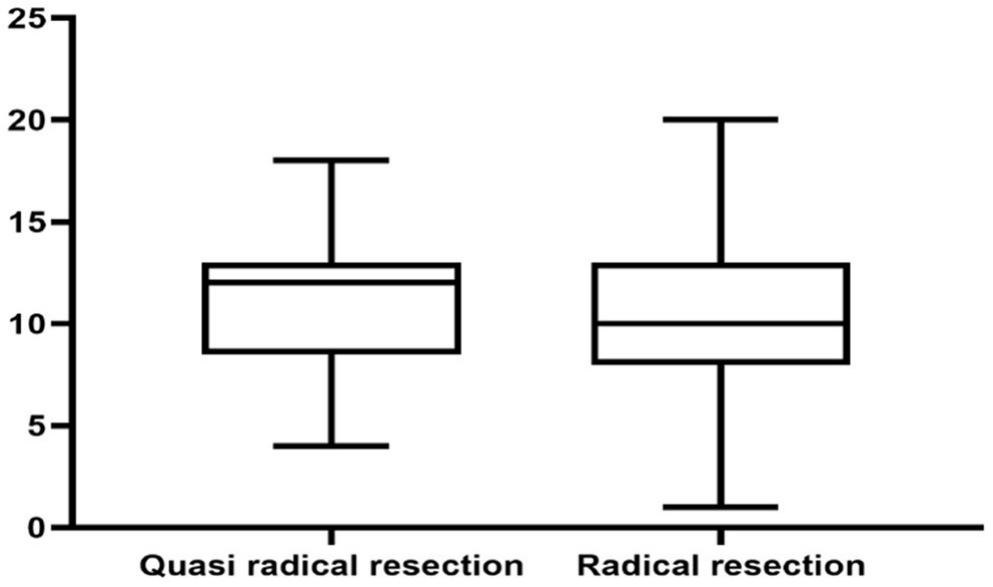
Physical domain.

**Figure 4:**
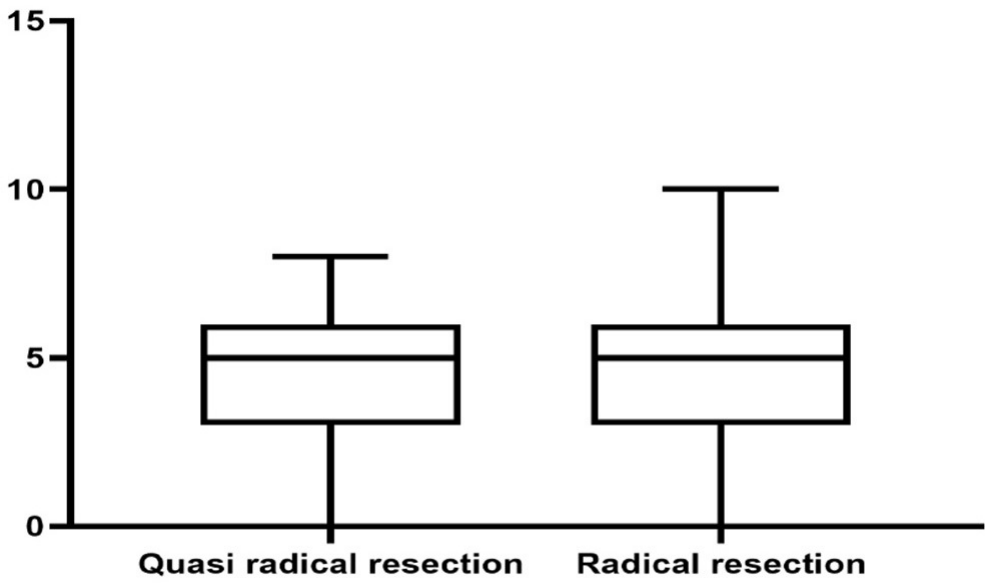
Psychological domain.

**Figure 5:**
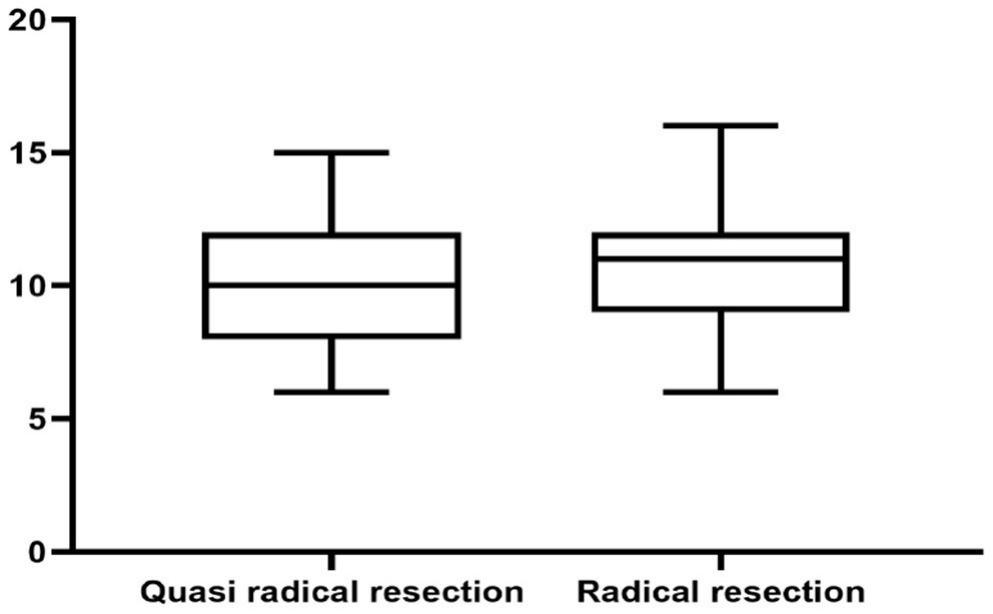
Independent domain.

**Figure 6:**
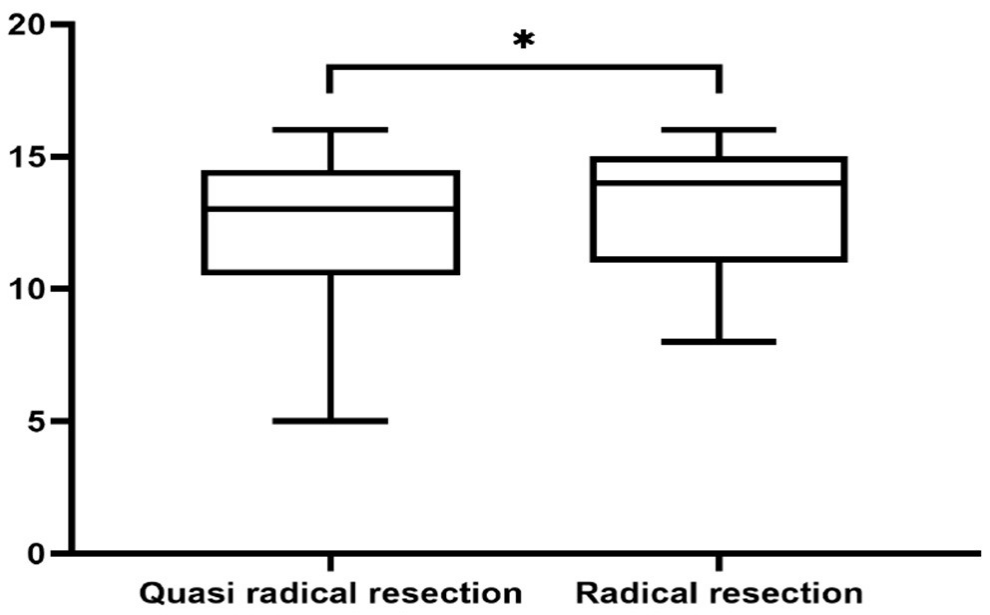
Social relations domain. *Indicates statistical differences between the two groups.

**Figure 7:**
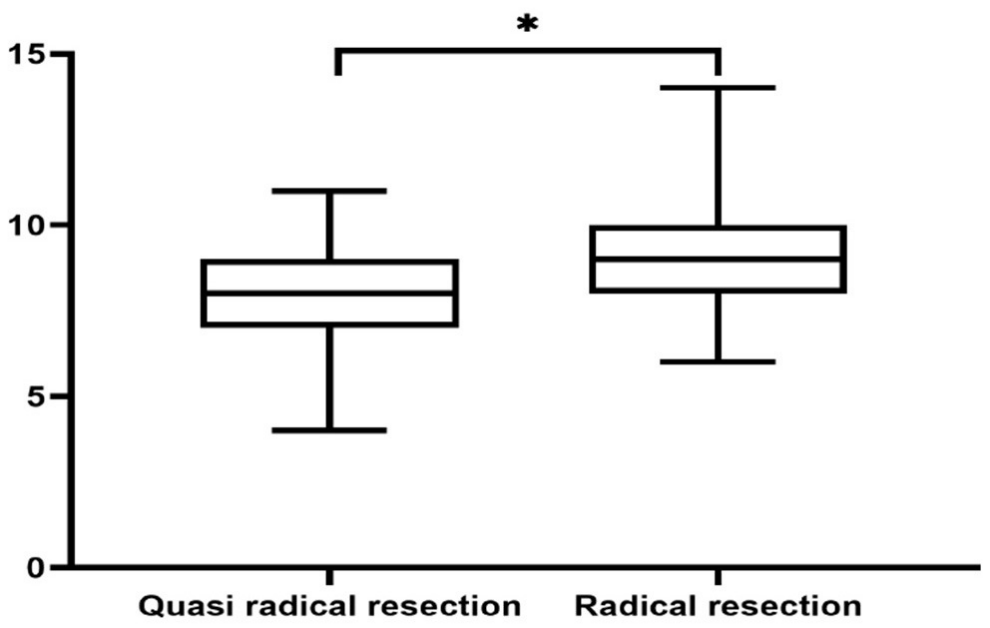
Environment domain. *Indicates statistical differences between the two groups.

There was no significant difference in survival curve analysis and total QoL score between the two groups (*P* > 0.05). The two groups were compared in five areas of QoL, among which there was no significant difference in physiological domain, psychological domain and independence domain (*P* > 0.05), and the difference was statistically significant between 5-year recurrence rate, social relationship domain and environment domain (*P* < 0.05), as shown in Figures 1–7 and Table 3.

**Table 3:** Comparison of quality of life scores and 5-year recurrence rate between the two groups.

**Indices**		**Quasi-radical group(*n* = 53)**	**Radical group(*n* = 51)**	***P***
Total quality of life score		46 ± 7	48 ± 8	0.207
Physiological domain score		12 (9, 13)	10 (8, 13)	0.442
Psychological domain score		5 (3, 6)	5 (3, 6)	0.483
Independence score		10 (8, 12)	11 (9, 12)	0.392
Social relationship score		13 (11, 15)	14 (11, 15)	0.040
Environmental domain score		8 (7, 9)	9 (8, 10)	0.001
5-year recurrence	Yes	13	4	0.021
	No	40	47	

## Discussion

With the continuous improvement of the global medical level, the restricted area of liver surgery technology has been gradually broken. The size of the lesion or the involvement of blood vessels or bile ducts are no longer contraindications for radical surgery for advanced HAE (19, 20). The 10–15 year mortality of patients with HAE without treatment after diagnosis is 90% to 100% (21, 22). After radical resection of HAE, the complication rate was significantly reduced and the survival time was significantly prolonged (23–25). It was also reported that Radical surgery for HAE can effectively improve the QoL of patients (26). However, the treatment of HAE on the Qinghai Tibet Plateau has its particularity (27). For example, the source of patients is basically Tibetan people in pastoral areas. Due to the influence of religious beliefs, family economy and other factors, most patients seek medical treatment with jaundice, and the course of disease is at an advanced stage. Moreover, most patients with advanced HAE cannot accept artificial materials (such as artificial blood vessels and diaphragmatic patches) replacement or liver transplantation. In addition, restricted by geographical environment, regional economic conditions and medical level, patients with advanced HAE have a lower rate of radical surgical treatment. Therefore, for such patients with advanced HAE who already have serious liver complications, conventional hepatectomy can not achieve the purpose of radical cure, and the patients refuse to undergo liver transplantation, in order to alleviate their pain and prolong their survival, we chose a controversial surgical method that was called quasi- radical lesion resection in China. However, through our postoperative follow-up of patients with advanced HAE, it is found that the efficacy of quasi-radical surgery combined with albendazole is no less than those of radical surgery only in terms of QoL of patients, the similar results have also been reported abroad (28).

This study showed that the patients in the quasi-radical group had more intraoperative bleeding and higher severity of postoperative complications. Studies on the relationship between preoperative Child-Pugh grade and postoperative complications have been reported that the complication rate of radical surgery is significantly lower than that of quasi-radical surgery (29). However, the results of this study showed that there was no significant difference in the total QoL score between the two types of surgery, indicating that the long-term efficacy of quasi-radical surgery is worth affirmatory. In order to further demonstrate the effectiveness of lesion resection in the treatment of advanced HAE, the author analyzed the cases after quasi-radical operation one by one, then found that quasi-radical lesion resection is not only in line with the principle of individualized treatment, but also in line with the development law of the author's liver surgery team. The specific reasons are as follows: Firstly, the object of this study is advanced HAE, which has the characteristics of large lesion volume, wide invasion range and a sea of liver complications. Secondly, more than 95% of the study population is Tibetan with devout religious beliefs, and most of the patients are extremely resistant to organ and tissue transplantation. Finally, the Qinghai Provincial Hydatid Diagnosis and Treatment Center treats about 250 patients with HAE every year, including about 150 cases of surgical treatment and less than 70 cases of radical lesion resection. Quasi-radical surgery is mainly concentrated in 2012–2016 year, which is the primary stage of the development of liver surgery technology for the author's team. The preoperative evaluation means of liver surgery are insufficient, and there are difficulties in the treatment of intrahepatic large vessels and bile ducts, resulting in a high incidence of intraoperative bleeding and bile duct injury. Therefore, under the influence of many factors mentioned above, in order to prolong the survival time of patients with advanced HAE, reduce the surgical risk and postoperative mortality, we can only choose quasi-radical lesion resection to replace radical lesion resection. Although some scholars have classified quasi-radical surgery as palliative surgery, according to the results of this study, the author believes that quasi-radical surgery should not be simply classified as palliative surgery. Although the negative margin could not be achieved by quasi-radical surgery, the residual lesions lost activity after high temperature burning. To some extent, quasi-radical surgery has also achieved the goal of radical lesion resection and most patients who underwent quasi-radical lesion resection have the same survival time and QoL as those who underwent radical lesion resection. However, we do not deny the advantages of radical surgery, because radical lesion resection is still the first choice for the treatment of advanced HAE. And quasi-radical surgery is only a second-best choice or the indications for surgery should be strictly controlled.

In conclusion, for patients with advanced HAE who cannot achieve radical lesion resection by conventional hepatectomy or are unwilling to accept liver transplantation, quasi-radical lesion resection may be the only choice to effectively prolong their survival time. However, there are still many deficiencies in this study, such as single center experience, small number of enrolled cases and short follow-up time. Therefore, we look forward to further communication and discussion on such clinical research in other Liver Hydatid Diagnosis and Treatment Centers.

## Data Availability Statement

The original contributions presented in the study are included in the article/supplementary material, further inquiries can be directed to the corresponding authors.

## Ethics Statement

The studies involving human participants were reviewed and approved by the Ethics Committee of the Qinghai Provincial People's Hospital. Written informed consent to participate in this study was provided by the participants' legal guardian/next of kin.

## Author Contributions

JA, SZ, and HW conceived and designed the study. JA, WG, JC, and XA collected the data. JA and WG contributed to data analysis, interpretation, and writing article. JY approved the study and this submission. All authors contributed to the article and approved the submitted version.

## Funding

This work was supported by Basic Research Project of Qinghai Province (No. 2020-wjzdx-27), Basic Research Project of Qinghai Province (No. 2022-0301-ZJC-0114), Basic Research Project of Qinghai Province (No. 2022-0301-ZJC-0051), Qinghai Clinical Medical Research Center (No. 2018-SF-L3), and Qinghai Province Key Laboratory of Laboratory Medicine.

## Conflict of Interest

The authors declare that the research was conducted in the absence of any commercial or financial relationships that could be construed as a potential conflict of interest.

## Publisher's Note

All claims expressed in this article are solely those of the authors and do not necessarily represent those of their affiliated organizations, or those of the publisher, the editors and the reviewers. Any product that may be evaluated in this article, or claim that may be made by its manufacturer, is not guaranteed or endorsed by the publisher.
