# Corrigendum: Study on the Effect of Quasi-Radical Lesion Resection on the Quality of Life of Patients With Advanced Hepatic Alveolar Echinococcosis

**DOI:** 10.3389/fsurg.2022.916596

**Published:** 2022-06-17

**Authors:** Jide A, Jinping Chai, Wenlu Guo, Shunyun Zhao, Hao Wang, Xiangren A, Jinyu Yang

**Affiliations:** ^1^ Medical College of Soochow University, Suzhou, China; ^2^ Department of Hepatic Hydatidosis, Qinghai Provincial People's Hospital, Xining, China; ^3^ Department of Internal Medicine-Cardiovascular, Qinghai Provincial People’s Hospital, Xining, China; ^4^ Qinghai Province Key Laboratory of Laboratory Medicine, Department of Clinical Laboratory, Qinghai Provincial People’s Hospital, Qinghai Clinical Medical Research Center, Xining, China; ^5^ Department of Hepatic Hydatidosis, Qinghai Provincial People’s Hospital, Xining, China; ^6^ Intensive Care Unit, Qinghai Provincial People,s Hospital, Xining, China

**Keywords:** hepatic alveolar echinococcosis, quasi radical lesion resection, radical lesion resection, quality of life, total survival time

**A Corrigendum on**

**Study on the Effect of Quasi-Radical Lesion Resection on the Quality of Life of Patients With Advanced Hepatic Alveolar Echinococcosis**

*by A, J., Chai, J., Guo, W., Zhao, S., Wang, H., A, X. and Yang, J. (2022). Front. Surg. 8:821373. doi: 10.3389/fsurg.2021.821373*

In the published article, there was an error in affiliation “1”. Instead of “Soochow University, Suzhou, China”, it should be “Medical College of Soochow University, Suzhou, China”. The authors apologize for this error and state that this does not change the scientific conclusions of the article in any way. The original article has been updated.

